# Optimisation of sugar and solid biofuel co-production from almond tree prunings by acid pretreatment and enzymatic hydrolysis

**DOI:** 10.1186/s40643-024-00743-x

**Published:** 2024-03-11

**Authors:** Manuel Cuevas-Aranda, Mª Lourdes Martínez-Cartas, Fahd Mnasser, Adnan Asad Karim, Sebastián Sánchez

**Affiliations:** 1 Department of Chemical, Environmental and Materials Engineering, University of Jaén, Avda. de La Universidad s/n, 23700 Linares, Spain; 2 Olive Grove and Olive Oil Research Institute, University of Jaén, Campus de Las Lagunillas s/n, 23071 Jaén, Spain

**Keywords:** Almond tree prunings, Acid hydrolysis, Enzymatic hydrolysis, Monosaccharides, Response surface methodology

## Abstract

Almond pruning biomass is an important agricultural residue that has been scarcely studied for the co-production of sugars and solid biofuels. In this work, the production of monosaccharides from almond prunings was optimised by a two-step process scheme: pretreatment with dilute sulphuric acid (0.025 M, at 185.9–214.1 ℃ for 0.8–9.2 min) followed by enzyme saccharification of the pretreated cellulose. The application of a response surface methodology enabled the mathematical modelling of the process, establishing pretreatment conditions to maximise both the amount of sugar in the acid prehydrolysate (23.4 kg/100 kg raw material, at 195.7 ℃ for 3.5 min) and the enzymatic digestibility of the pretreated cellulose (45.4%, at 210.0 ℃ for 8.0 min). The highest overall sugar yield (36.8 kg/100 kg raw material, equivalent to 64.3% of all sugars in the feedstock) was obtained with a pretreatment carried out at 197.0 ℃ for 4.0 min. Under these conditions, moreover, the final solids showed better properties for thermochemical utilisation (22.0 MJ/kg heating value, 0.87% ash content, and 72.1 mg/g moisture adsorption capacity) compared to those of the original prunings.

**Graphical Abstract:**

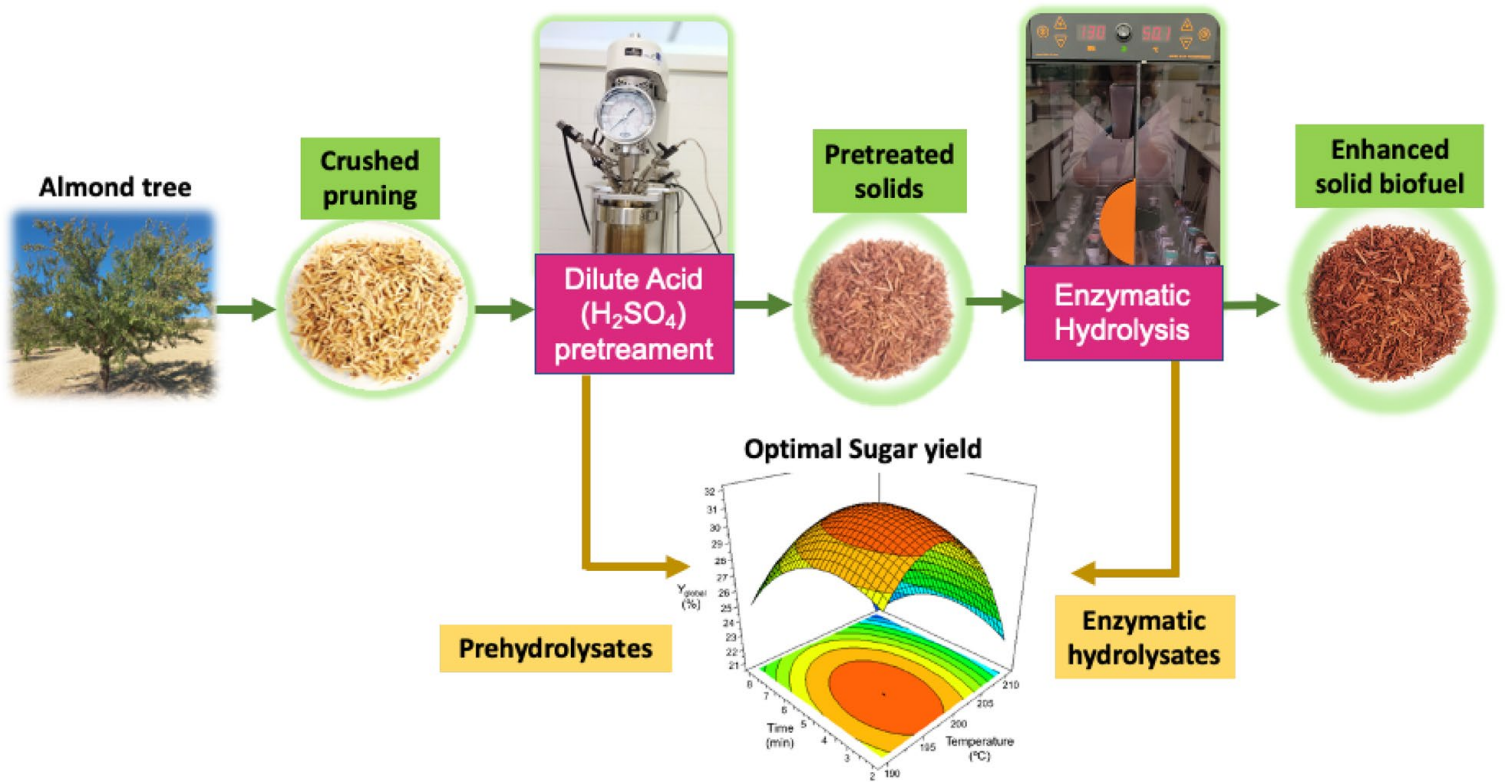

## Introduction

The transition from a fossil fuel-based economy to a bioeconomy, utilizing a biomass-biorefinery model, (is vital for sustainable development and a safer environment (Popp et al. 2021; Voogt et al. 2023). In this context, lignocellulosic biomass (e.g., agricultural residues) can be used for diverse applications such as renewable energy generation (Valizadeh et al. 2022; Naqvi et al. 2023) or the production of monosaccharides and others industrial chemicals (Zheng et al. 2022; Sekyere et al. 2023). Monosaccharides or single sugars (e.g., glucose, xylose arabinose) are a group of renewable chemical compounds that can be utilised as precursors for the industrial production of biofuels (Heinonen and Sainio 2013), 2,5-dimethylfuran (Lim and Rashidi 2023) and petrochemicals such as isobutene (Van Leeuwen et al. 2012). But the lignocellulosic biomass naturally contains polysaccharides (mainly cellulose and hemicellulose), which need to be cleaved into monosaccharides. Furthermore, the recalcitrant nature of these polysaccharides and their structural integration with lignin pose a significant challenge to the production of monosaccharides (Hu et al. 2023). Acid and enzymatic hydrolyses of lignocellulosic biomass are commonly used for the production of monosaccharides. Various acids, such as sulphuric acid (predominantly used), hydrochloric acid, acetic acid, and phosphoric acid have been reported for hydrolysis. The use of concentrated acids poses several drawbacks. The high corrosiveness of the acidic medium necessitates that the reactors be made with expensive materials (such as acid-resistant alloys or ceramics). In addition, large quantities of chemicals are required for the neutralisation of the acid hydrolysate, generating salts whose management increases the cost of the process (Jung and Kim 2015). Using diluted acids at high temperatures (120–260 ℃) for short reaction times (less than 10 min) is an alternative that could alleviate those issues and has been widely employed in the hydrolysis of polysaccharides (Shahbazi and Zhang 2010; Heinonen and Sainio 2013). Enzymatic hydrolysis, mainly using cellulase enzyme,) for the selective conversion of cellulosic fraction into monosaccharides has also been widely reported, but it has the disadvantage of being a slower (48–84 h) process compared to acid hydrolysis. This is due to the structural complexity of biomass and the limited access of the cellulase enzyme to the cellulose (Arantes and Saddler 2010). To resolve the aforementioned issues, the pretreatment of lignocellulosic biomass with dilute acid at high temperature and subsequent enzymatic hydrolysis of pretreated solids is reported to be a promising integrated process for the production of monosaccharides (Solarte-Toro et al. 2020; Saini et al. 2022). The acid pretreatment of biomass cleaves the hemicellulose chains to produce a prehydrolysate with significant amounts of monosaccharides and a solid fraction with a more exposed cellulosic fraction that can be hydrolysed by cellulases (Kabel et al. 2007). Optimisation of the pretreatment conditions, specifically temperature and reaction time, has been carried out to maximise the yield of sugar. Appropriate pretreatment conditions are required because mild treatment conditions limit the conversion of hemicellulose to monosaccharides, preserving some hemicellulosic structures that act as barriers to the action of cellulases, whereas harsher conditions cause the transformation of hemicellulose sugars to degradation products such as furfural and 5-hydroxymethylfurfural (Montané et al. 2002). In addition, pretreatment conditions may modify the structure of lignin, which would subsequently affect the non-productive adsorption of cellulases onto pseudo-lignin (Yuan et al. 2021). Some authors have pointed out that pretreatment conditions leading to maximum hemicellulose sugar yields (via acid pretreatment) and glucose (via enzymatic hydrolysis of pretreated cellulose) are different. For instance, pretreating olive tree pruning with sulphuric acid for 10 min resulted in the highest recovery of hemicellulose sugars (83%) at 170 ℃ with a catalyst concentration of 1%, the maximum enzymatic digestibility (76.5%) at 210 ℃-1.4%, and the maximum total sugar yield (75%) at 180 ℃-1% (Cara et al. 2008). The Response Surface Methodology (RSM) is a mathematical and statistical approach utilised for modelling and analysing problems wherein responses are influenced by multiple variables (or factors). The primary goal of applying RSM is to identify the values of the variables that lead to optimal responses using a minimum number of experiments. RSM has been applied to know the optimal conditions for obtaining monosaccharides from the acid hydrolysis of different lignocellulosic biomasses, such as olive stone (Saleh et al. 2014), olive tree biomass (García Martín et al. 2013; Yildirim et al. 2022), acacia wood (Lee and Yu 2020), date seeds (Hasan Ba Hamid and Ku Ismail 2020), coconut coir (Gundupalli and Bhattacharyya 2019), etc. The pretreatment conditions applied to lignocellulosic biomasses are strongly affected by biomass characteristics, so optimising the processes for each biomass type is very important (Shahbazi and Zhang 2010).

Almond tree (*Prunus dulcis* (Mill) D. A. Webb) is one of the woody crops whose production has experienced greater growth in recent decades worldwide. For instance, in 2021 the harvested area and shelled almond production were 2.28 × 10^6^ ha and 3.99 × 10^6^ tons, respectively. This represents an increase of 37% in the harvested area and 175% in fruit production compared to 2000 (FAOSTAT 2023). These percentages could be explained by the human health benefits of almonds and their derived products (Johnston et al. 2017; Barreca et al. 2020). Globally, Spain holds first rank in terms of almond tree harvested area (744,000 hectares) and is the second-largest producer of almonds, only behind the US (MAPA 2022). The pruning of almond tree branches is an annual operation that is carried out to maintain the health and productivity of the trees, generating significant amounts of residual lignocellulosic biomass (Velázquez-Martí et al. 2011). However, research studies on the valorisation of almond-tree pruning biomass are scarce. In an earlier work of our research group (Cuevas et al. 2014), different pretreatments (liquid hot water, diluted sulphuric acid and delignification with hydrogen peroxide) were combined with enzymatic hydrolysis for sugar production from almond tree prunings. This study concluded that pretreatment with dilute sulphuric acid was the most suitable, considering both the sugar yield and the simplicity of the process. Nevertheless, in this study, the conditions for acid pretreatment were not optimised using response surface methodology, and the co-production of solid biofuels and their characteristics were not included. This analysis would be of great interest to valorise the final lignin-rich solid by thermochemical routes such as combustion, gasification or pyrolysis (Woytiuk et al. 2017; Liu et al. 2022; Ma et al. 2022). The main objective of the present study was to investigate the potential of almond prunings for sugar production through a two-stage sequential process (pretreatment with dilute sulphuric acid and enzymatic hydrolysis of the pretreated solids) optimising the pretreatment conditions. The acid pretreatment was performed based on a response surface methodology, for the determination of optimal conditions to maximise both sugar recovery in the prehydrolysate and glucose production through the enzymatic hydrolysis of pretreated cellulose. The ultimate goal was to identify the pretreatment that maximises overall sugar production. The influence of process conditions is also being studied to co-produce a solid lignin-rich material with improved energy characteristics (heating value, ash content and moisture adsorption capacity). The present research results and findings will contribute towards promoting the use of almond tree pruning biomass as a substrate in lignocellulosic biomass-based biorefinery.

## Materials and methods

### Raw material

The almond prunings of *Prunus dulcis* (Mill) D. A. Webb cv. "Tuono" was collected from a farm located in Alhama de Granada (province of Granada, Spain, UTM coordinates 37°01ʹ46.53″N, 3°56ʹ16.64″W). Pruning biomass was air dried in the laboratory to reach an equilibrium humidity of 7.05 ± 0.30%. Subsequently, the biomass was crushed in a knife mill (Retsch GMBH, Germany) and sieved to prepare two different particle sizes of 0.125–0.300 mm and 0.425–2 mm. Sizes smaller than 0.125 mm were discarded because they contained the highest proportion of inorganic matter during sieving. The fraction 0.125–0.300 mm was reserved for the chemical characterisation of the original material, which requires small particle diameters to ensure complete chemical attack of the substrate. This same particle size range (0.125–0.300 mm) was employed in a previous study for the characterisation of almond prunings (Cuevas et al. 2014). Dilute acid pretreatments were applied to biomass with particle sizes in the range of 0.425–2 mm, a size sufficiently small to ensure that the solid diameter does not limit the attack suffered by the material. In this regard, some authors indicated that woody biomass fragments below 3 mm usually exhibit the same conversion when subjected to dilute acid pretreatments (Vidal et al. 2011). The two batches of biomass were homogenised to prepare representative samples and stored in a dry place.

### Pretreatment with diluted sulphuric acid

The pretreatment was carried out in a 2 dm^3^ Parr Series 4522 reactor (Moline, IL, USA) equipped with an internal protective vessel constructed of borosilicate glass. The reactor was charged with 107.5 *g* of raw material (equivalent to 100 *g* of dry solid) and 1000 cm^3^ of dilute (0.025 M) sulphuric acid solution to work with a solid/liquid ratio equal to 1/10.

**Fig. 1:**
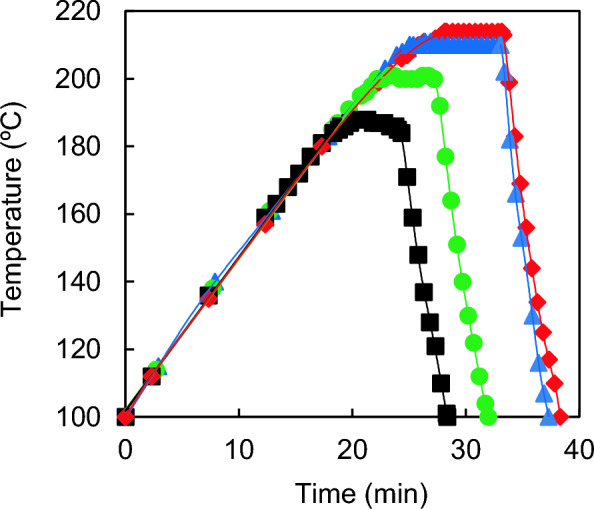
Temperature profile of four acid pretreatments:  214.1 ℃-5 min;  210 ℃-8 min;  200 ℃-5 min;  185.9 ℃-5 min

The suspension was stirred at 250 rpm and heated at a rate of 4.4 ± 0.2 ℃/min until reaching an established temperature (*T*_*R*_, ºC), followed by maintaining this temperature for a certain period or “reaction time” (*t*_*R*_, min). Finally, the cooling stage of the reactor was carried out by circulating cold water through an internal coil. Experiments were conducted at varied temperatures (*T*_*R*_: 185.9–214.1 ℃) and reaction times (*t*_*R*_: 0.8–9.2 min). These experimental conditions were chosen based on previous results regarding the dilute acid hydrolysis of almond tree prunings (Cuevas et al. 2014), where reaction temperatures in the range of 180–230 ℃ with a constant reaction time (*t*_*R*_: 5 min) were employed). This study established that the optimal *T*_*R*_ values for the recovery of hemicellulosic sugars in the prehydrolysate and for the enzymatic hydrolysis of pretreated cellulose were 190 ℃ and 230 ℃, respectively, while a temperature of 200 ℃ was most suitable for achieving maximum sugar recovery considering both pretreatment and enzymatic hydrolysis stages together. The temperature profiles of four pretreatments experiments are shown in Fig. 1. After cooling to room temperature, the reactor was opened and its contents were vacuum filtered to recover two fractions: water-insoluble solids and liquor (or prehydrolysate). The solid fraction was washed repeatedly with distilled water until the prehydrolysate reached a final volume of 2 dm^3^. This liquid was analysed for the determination of simple sugars and 5-hydroxymethylfurfural contents. The pretreated-washed solids (or washed insoluble solids, WIS) were dried at room temperature (20 ℃) and stored for subsequent chemical composition analysis and enzymatic hydrolysis experiments.

### Experimental design

An experimental design based on the Central Composite Circumscribed (CCC) type was selected for acid pretreatments with two factors (temperature-*T*_*R*_, and reaction time-*t*_*R*_) having two levels. The design includes four factorial points, four axial points, and three points at the centre of the experimental domain (Table 1). In the response surface design for each factor, a low level (– 1) and a high level (+ 1) are defined. The center point is coded to 0, while axial points (or star points) are located at a given distance α = 1.41 from the design center in each direction on each axis to allow estimation of curvature. The experimental data analysis was performed using Modde 6.0 statistical software (Umetric AB, Umeå, Sweden). Equation 1, obtained by regression analysis, was followed to study each response.

1 $$Y = \,\, a_{0} + (a_{1}\cdot T_{R}) + (a_{2}\cdot t_{R}) + (a_{3}\cdot T_{R}\cdot T_{R}) + (a_{4}\cdot t_{R}\cdot t_{R}) + (a_{5}\cdot T_{R}\cdot t_{R})$$

where Y is the response value, *T*_R_ and *t*_R_ are the coded values of the two independent variables, and a_i_ are the intercept term (a_0_), the linear effects (a_1_ and a_2_), the squared effects (a_3_ and a_4_), and the interaction effect (a_5_) calculated from the experimental data.

**Table 1:** Operational conditions assayed for sulphuric acid pretreatment of almond tree pruning

Pretreatment code	*T*_R_ (ºC)		*t*_R_ (min)	
	Coded	Real	Coded	Real
P1	0	185.9	− 1.41	5.0
P2	− 1	190.0	− 1	2.0
P3	− 1.41	200.0	0	0.8
P4	+ 1	190.0	− 1	8.0
P5	0	200.0	0	5.0
P6	0	200.0	0	5.0
P7	0	200.0	0	5.0
P8	− 1	210.0	1	2.0
P9	+ 1.41	200.0	0	9.2
P10	+ 1	210.0	+ 1	8.0
P11	0	214.1	+ 1.41	5.0

*T*_R_: reaction temperature. *t*_R_: reaction time (holding time at *T*_R_)

### Enzymatic hydrolysis

The raw material and water-insoluble solids obtained from acid pretreatments (WIS) were subjected to enzymatic hydrolysis using a cellulolytic complex (Celluclast 1.5 L) which showed an activity of 33.04 FPU/cm^3^ (Ghose 1987). Enzyme loads tested were 10 FPU/*g* solid and 15 FPU/*g* solid. For some experiments, a fungal β-glucosidase (Novozyme 188) was used in order to reduce inhibition by cellobiose. The load of β-glucosidase was 30 International Units (IU) per gram of solid. To perform the calculations, solid weights were determined on a dry basis. Chloramphenicol was added at a final concentration of 50 μg/cm^3^ to prevent microbial growth and consumption of the released sugars. Enzymatic hydrolyses were performed in 0.05 M sodium citrate buffer (pH 4.8) at 50 ℃ on a rotary shaker (HT-Ecotron benchtop incubator) at 150 rpm for 96 h and 10% (w/v) solid substrate concentration (5 *g*/50 cm^3^). Samples were withdrawn from the reaction media at different times (24, 48, 72, and 96 h) to determine the concentration of glucose and total reducing sugars (TRS). From these values, enzyme digestibility (*ED*), glucose yield (*Y*_Glu/RM_), and total reducing sugars yield (*Y*_TRS/RM_) were calculated by applying Eqs. 2–4.

2 $$ED (\%) = \, grams \, of \, glucose \, by \, enzymatic \, hydrolysis/100 \, g \, glucose \, in \, the \, substrate$$

3 $$Y_{Glu/RM} = \, grams \, of \, glucose \, by \, enzymatic \, hydrolysis/100 \, g \, raw \, material$$

4 $$Y_{TRS/RM} = \, grams \, of \, total \, reducing \, sugars \, by \, enzymatic \, hydrolysis/100 \, g \, raw \, material$$

The enzymes were procured from Novo Nordisk Bioindustrial (Madrid, Spain), and all assays were performed in duplicate.

### Analytical methods

The protocol described by Cuevas et al. (2014) was followed for the raw material characterisation. In the case of the pretreated solids (WIS), the TAPPI methods T12 os-75 and T15 os-58 were used to determine moisture and ash contents, respectively, whereas the methodology described by Saleh et al. (2014) was used to measure the amount of cellulose, hemicellulose and acid insoluble lignin (AIL). To determine the concentration of monosaccharides (glucose, xylose, arabinose, galactose, and mannose) and 5-hydroxy-methyl-furfural in prehydrolysates, liquors were diluted and filtered through a 0.22 mm nylon membrane (Millipore). Thereafter, these samples were injected into a Dionex ICS-3000 chromatography system (Dionex Corporation, Sunnyvale, CA, USA) equipped with a CarboPacTM PA20 (3 × 150 mm) analytical column, a CarboPacTM PA20 (3 × 30 mm) guard column, and a pulsed amperometric detector. The eluent was NaOH (2 mM), and elution took place at 30 ℃ using a flow rate of 1 cm^3^/min. Glucose and total reducing sugar (TRS) concentrations from enzymatic hydrolysis samples were measured by an enzymatic glucose assay kit (Chemelex LABKIT Glucose-TR, Barcelona, Spain) and the DNS method (Miller 1959), respectively. The determination of the higher heating value (HHV) of solids (raw material, pretreated solids, and solids resulting from enzymatic hydrolysis) was carried out using a Parr automatic isoperibol calorimeter (series 6400) according to ISO 18125:2017. The equilibrium moisture contents of solids were determined by a method previously described (Aguado et al. 2020) using an oversaturated solution of sodium chloride at 20 ℃, which provides an equilibrium relative humidity of 75.5% in the surrounding air (Greenspan 1977). All assays were performed in duplicate.

## Results and discussion

### Effect of pretreatment conditions on the fractionation of almond prunings

The raw material had the following composition on a dry basis: 31.9 ± 0.5% cellulose, 22.1 ± 1.0% hemicelluloses, 26.5 ± 1.4% acid insoluble lignin, 14.2 ± 0.7% water extractives, 3.4 ± 0.4% organic extractives, and 1.28 ± 0.04% ash. From 100 *g* of dry raw material, hydrolysis of the hemicellulosic fraction could theoretically generate 16.6 *g* of xylose, 2.5 *g* of arabinose, 1.4 *g* of galactose, and 1.3 *g* of mannose. The theoretical maximum amount of glucose obtainable from 100 *g* of dry raw material would be 35.4 *g*. These data agree with a previous study on almond prunings (Cuevas et al. 2014), which reported contents of cellulose, hemicelluloses and acid insoluble lignin of 31.3%, 23.0%, and 28.7%, respectively. It is known that diluted sulphuric acid, acting at high temperatures and for short periods of time, can cause a significant modification in the chemical composition of lignocellulosic biomasses. Typically, the acid catalyst induces a strong hydrolysis of hemicellulose and partial depolymerisation of cellulose. This can be explained by the higher crystallinity of cellulose, which reduces the penetration of chemical agents into its structure (Zhou et al. 2021). Regarding the lignin fraction, effective removal of this aromatic biopolymer is difficult using a dilute acid pretreatment because, under some operational conditions, the rate of depolymerisation is slower than repolymerisation, leading even to an elevation in the molecular weight of the lignin compared to its native state (He et al. 2022). In any case, the hydrolysis of polysaccharides will lead to a pronounced decrease in the recovery of the solid fraction (quantified as “Total Gravimetric Recovery”, or TGR) after acid treatment. When almond pruning was pretreated with 0.025 M sulphuric acid under the operating conditions specified in Table 1, the TGR values ranged from 54.7% to 60.1%, corresponding to pretreatments P11 (214.1 ℃-5 min) and P2 (190 ℃-2 min), respectively (Table 2). The small variation in the amount of solid recovered can be due to the application of a narrow temperature range (185.9 ℃–214.1 ℃) and short reaction times (0.8 min–9.2 min). Nevertheless, it is observed that, in general, elevating the severity of the pretreatment (higher temperatures and reaction times) resulted in a discernible reduction in the TGR values. Table 2 also shows that all pretreatments produced a strong reduction in hemicellulose content. Thus, the pretreatment performed at 185.9 ℃-5 min (P1) generated WIS with 3.4% hemicellulose. This means that only 9.4% of the original polymer was maintained, while in the solids pretreated in the P5-P11 assays, the hemicellulose contents were less than 1.5%. These values are similar to those reported by Kabel et al. (2007) when they achieved 8% residual xylans by hydrolysing wheat straw with sulphuric acid (2.5%) at 170 ℃ for 15 min. The strong loss of hemicellulose during pretreatment caused the percentages of cellulose in the WIS (41.0–47.1%) to be clearly higher than those of the raw material (31.9%). However, the acid attack also caused some loss of cellulose, whose conversion was increased under the most severe pretreatment conditions. Thus, while P1 pretreatment (185.9 ℃-5 min) only eliminated 11.3% cellulose, P10 pretreatment (210.0 ℃-8.0 min) resulted in a 28.8% biopolymer conversion (Table 2). This same behaviour was described in a previous work where the same type of biomass was subjected to acid hydrolysis (Cuevas et al. 2014). Acid insoluble lignin was the structural material that underwent the least variation during pretreatment. It ranges from 38.1–41.1% in the WIS, with conversions in the range of 13.2–17.2%.

**Table 2:** Total gravimetric recovery (TGR) and composition of water insoluble solids resulting from sulphuric acid pretreatments^a^

Run	*T*_R_ (ºC)	*t*_R_ (min)	TGR (%)	Hemicellulose (%)	Cellulose (%)	Acid insoluble lignin (%)
P1	185.9	5.0	60.0	3.4 (90.6)^b^	47.1 (11.3)	38.1 (13.8)
P2	190.0	2.0	60.1	2.9 (92.0)	46.8 (11.8)	38.3 (13.2)
P3	200.0	0.8	59.2	2.5 (93.2)	43.5 (19.2)	38.4 (14.1)
P4	190.0	8.0	57.8	1.5 (96.2)	45.1 (18.2)	38.6 (15.7)
P5	200.0	5.0	57.2	1.4 (96.3)	43.9 (21.2)	39.0 (15.8)
P6	200.0	5.0	57.0	1.0 (97.4)	44.8 (19.9)	38.9 (16.3)
P7	200.0	5.0	57.3	1.2 (96.9)	43.3 (22.2)	39.0 (15.8)
P8	210.0	2.0	56.6	0.0 (100.0)	45.0 (20.2)	39.2 (16.2)
P9	200.0	9.2	55.9	0.2 (99.6)	45.4 (20.5)	39.3 (17.2)
P10	210.0	8.0	55.4	1.3 (96.8)	41.0 (28.8)	41.1 (14.2)
P11	214.1	5.0	54.7	0.0 (100.0)	44.1 (24.3)	41.1 (15.2)

^a^Percentages expressed on a dry basis

^b^The percentage of biopolymer (hemicellulose, cellulose, or acid insoluble lignin) transformed during pretreatments is shown in brackets

The acid pretreatment of hemicellulose and cellulose in almond prunings produced a prehydrolysate with varied amounts of simple sugars. Table 3 shows the yields of glucose, xylose, arabinose, and the sum of mannose plus galactose for the eleven prehydrolysates. In addition, the total monomeric sugar (TMS) yield, calculated as the sum of the yields of the five monosaccharides analysed, is incorporated. Xylose, glucose and arabinose were the most abundant monosaccharides in prehydrolysates, reaching yields of 4.87–14.50 *g*, 1.33–5.23 *g*, and 0.89–2.50 *g* per 100 *g* of dry raw material, respectively. The yield of galactose and mannose was very low for all operating conditions, and these compounds were not detected in the P10 and P11 tests. The TMS yield ranged from 7.38 *g* (P11) to 22.50 *g* (P6) per 100 *g* of dry raw material. For all monosaccharides, maximum yields were obtained with intermediate severity pretreatments, which led to a high generation of simple sugars (by hydrolysis of polysaccharides) while controlling losses of these monosaccharides by thermal degradation (to products such as 5-hydroxy-methyl-furfural). Concerning the monosaccharide degradation products, the P11 pretreatment, performed at the maximum temperature tested (214.1 ℃), resulted in a relatively low yield of 5-hydroxy-methyl-furfural (0.45 *g*/100 *g* dry raw material).

**Table 3:** Products yields (as *g*/100 *g* dry raw material) in the prehydrolysates obtained at different pretreatment conditions

Run	*Y*_glucose_	*Y*_xylose_	*Y*_arabinose_	*Y*_GAL+MAN_	*Y*_TMS_
P1	3.91	11.59	1.83	0.74	18.06
P2	4.51	13.43	1.45	1.66	21.06
P3	5.23	13.02	0.92	2.33	21.49
P4	3.78	11.13	2.02	0.40	17.34
P5	4.17	14.50	2.50	0.70	21.87
P6	4.88	14.34	2.28	1.00	22.50
P7	4.72	13.82	2.48	0.63	21.65
P8	2.34	8.20	0.89	0.89	12.32
P9	3.59	8.50	1.89	0.03	14.01
P10	2.29	5.42	1.43	nd	9.14
P11	1.33	4.87	1.17	nd	7.38

GAL + MAN: galactose plus mannose. TMS: Total Monomeric Sugars. nd: not detected

Data from Tables 1 and 2 was analysed in the Modde 6.0 program to determine the effect of temperature (*T*_R_) and reaction time (*t*_R_) during the acid pretreatment of the almond pruning biomass. Mathematical models with statistical validity were checked to obtain the following responses: TGR for the pretreated solids; and xylose, glucose, arabinose and total monomeric sugar yields (*Y*_xylose_, *Y*_glucose_, *Y*_arabinose_ and *Y*_TMS_, respectively) for the prehydrolysates. The model coefficients (a_i_ in Eq. 1) were obtained from the ANOVA analysis along with the standard deviations for the different responses (Table 4). For all models, correlation coefficients (*R*^*2*^ and *R*^*2*^_adjust_) were acceptable. The value of *R*^2^ was 0.934 in the most unfavourable case (*Y*_glucose_), implying that only 6.6% of the total variations in the response were not explained by the model.

**Table 4:** Model parameters (a_i_), standard errors (SE), and significance level (*p*) for the mathematical models

Response variable	a_i_	SE	*p*-value (Prob > *F*)	*R*^2^	*R*^2^_adjust_
*TGR*, %	a_0_: 57.380	0.109	1.96·10^–19^	0.967	0.959
	a_1_: –1.679	0.128	1.10·10^–6^		
	a_2_: –1.036	0.128	4.08·10^–5^		
*Y*_glucose_^(1)^	a_0_: 4.438	0.159	1.91·10^–8^	0.934	0.906
	a_1_: –0.913	0.133	2.43·10^–4^		
	a_2_: –0.386	0.133	2.31·10^–2^		
	a_3_: –1.007	0.152	2.95·10^–4^		
*Y*_xylose_^(1)^	a_0_: 14.22	0.210	6.94·10^–10^	0.994	0.989
	a_1_: –2.556	0.128	1.05·10^–6^		
	a_2_: –1.434	0.128	3.09·10^–5^		
	a_3_: –2.982	0.153	1.18·10^–6^		
	a_5_: –1.717	0.153	2.98·10^–5^		
*Y*_arabinose_^(1)^	a_0_: 2.420	0.050	4.98·10^–9^	0.987	0.978
	a_1_: –0.260	0.030	1.38·10^–4^		
	a_2_: 0.310	0.030	5.14·10^–5^		
	a_3_: –0.461	0.036	1.43·10^–5^		
	a_5_: –0.509	0.036	8.05·10^–6^		
*Y*_TMS_^(1)^	a_0_: 22.007	0.389	2.04·10^–9^	0.991	0.985
	a_1_: –4.006	0.238	2.81·10^–6^		
	a_2_: –2.185	0.238	9.40·10^–5^		
	a_3_: –4.712	0.283	3.01·10^–6^		
	a_4_: –2.196	0.283	2.42·10^–4^		

Total gravimetric recovery (TGR), glucose yield (*Y*_Glucose_), xylose yield (*Y*_Xylose_), arabinose yield (*Y*_Arabinose_) and total monomeric sugars yield (*Y*_TMS_) for the sulphuric acid pretreatment of almond tree prunings

Significance level was defined as *p* < 0.05

^(1)^ Products yields are expressed as grams of product per 100 *g* of dry raw material

**Fig. 2:**
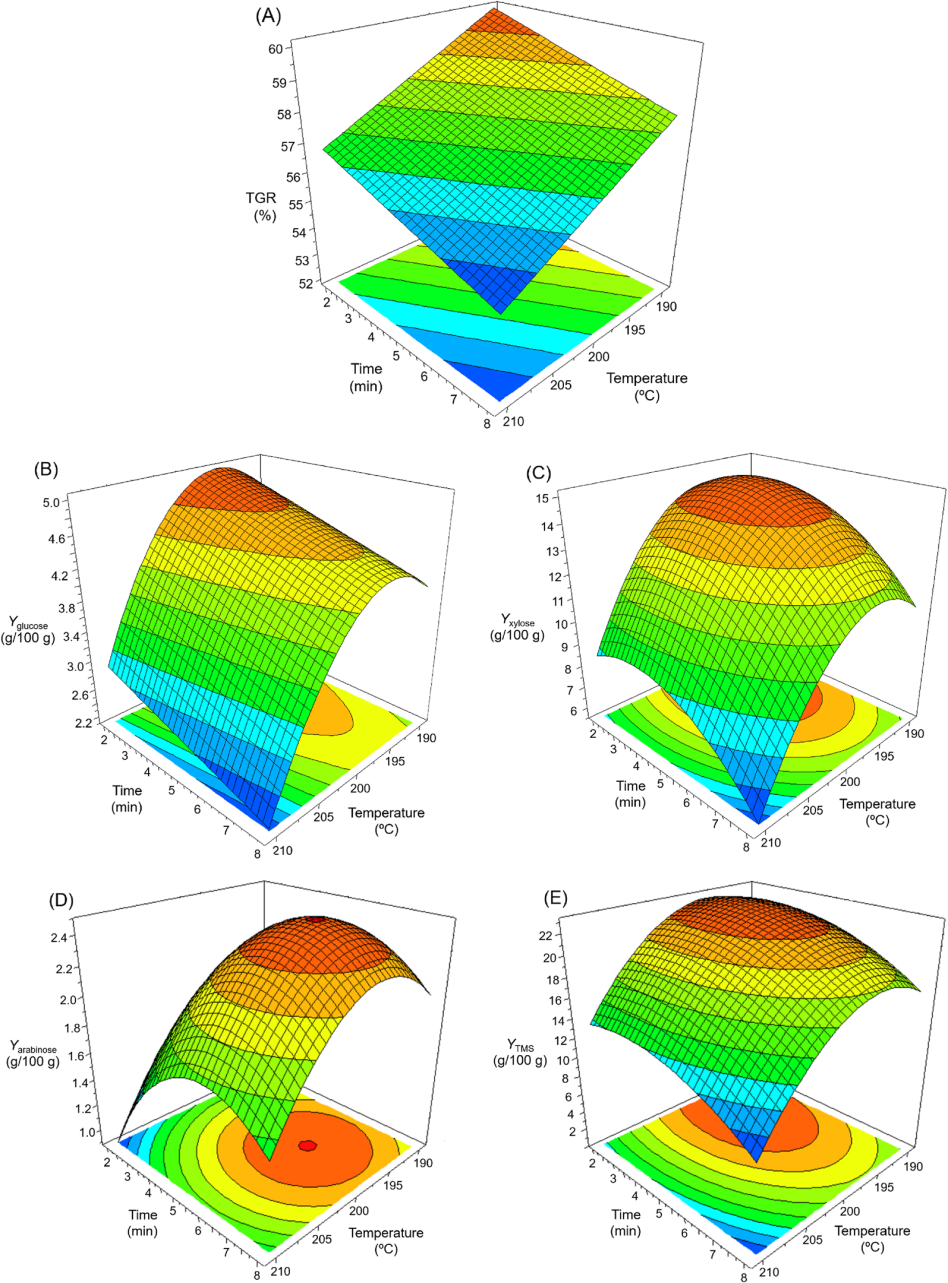
Response surface plots representing the effect of temperature and pretreatment time on TGR for WIS (**A**), and glucose yield (**B**), xylose yield (**C**), arabinose yield (**D**) and total monomeric sugar yield (**E**) in the prehydrolysates

The parameters given in Table 4 were used to represent the response surface plots shown in Fig. 2. For total gravimetric recovery (Fig. 2A), it was observed that both the temperature and the reaction time exerted a negative and linear effect on the response, with no quadratic terms and no interaction terms between the two factors. The same behaviour was earlier reported for the diluted acid pretreatment of olive stones at high temperatures (Saleh et al. 2014). In the case of sugar production, the glucose yield depended more on the temperature than the reaction time, reaching its maximum value at 195.4 ℃ for a fixed time (Fig. 2B). This could be explained by considering that below 195.4 ℃ no significant hydrolysis of cellulose occurred, whereas above that temperature, the rate of glucose degradation exceeds the rate at which the monosaccharide is obtained. The model predicts a maximum value for *Y*_glucose_ equal to 5.03 *g*/100 *g* dry raw material under the conditions of 195.4 ℃-2.0 min. Maximum yields of xylose (15.07 ± 1.3 *g*/100 *g* dry raw material), arabinose (2.50 ± 0.2 *g*/100 *g* dry raw material), and TMS (23.4 ± 2.1 *g*/100 *g* dry raw material) were reached under the conditions of 195.7 ℃-3.8 min, 197.1 ℃-5.9 min, and 195.7–3.5 min, respectively (Fig. 2C, D and E). Therefore, the pretreatment allowed 90.8% extraction of the xylose present in almond tree wood. This value is in close range to the data reported by different authors for the acid treatment of different biomasses: 89.7% for olive stone at 195 ℃ for 5 min (Saleh et al. 2014), 90.95% for pinewood at 106.7 ℃ for 4.57 h (Cao et al. 2018), and 94% for giant reed at 141.6 ℃ for 36.4 min (Shatalov and Pereira 2012). The previous bibliographic references reveal how maximum xylose extraction can be reached through an appropriate combination of reaction times and temperatures.

### Effect of pretreatment conditions on the enzymatic hydrolysis

**Fig. 3:**
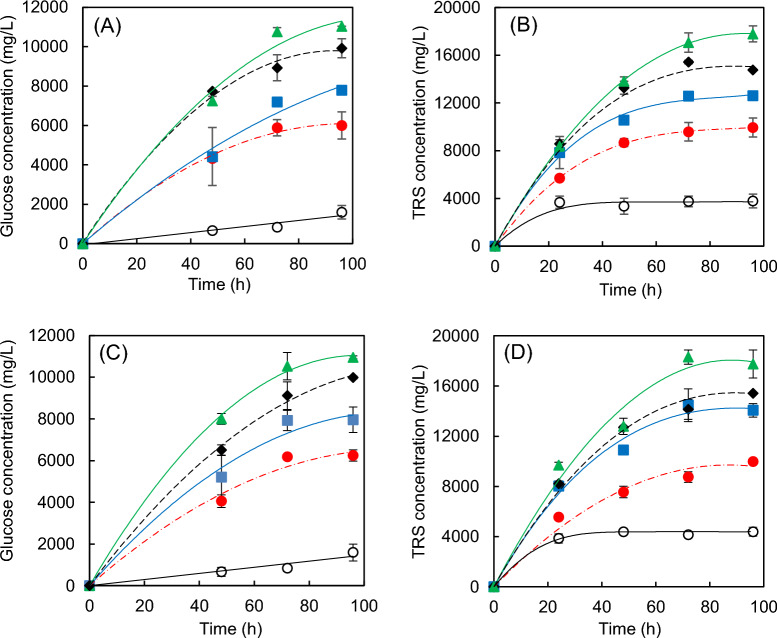
Time course of glucose and total reducing sugars (TRS) concentrations during enzymatic hydrolysis carried out at 10 FPU/*g* substrate (**a**,**b**) and 15 FPU/*g* substrate (**c**,**d**). Substrates: raw material (〇) and WIS obtained at 190 ℃-2 min (), 200 ℃-5 min (), 210 ℃-8 min (), and 214.1 ℃-5 min ()

The application of the cellulolytic complex "Celluclast 1.5 L" on pretreated almond prunings, employing two biocatalyst loads (10 FPU/*g* WIS or 15 FPU/*g* WIS), resulted in the production of both glucose and total reducing sugars (TRS), with stable concentrations achieved at 96 h (Fig. 3). It is important to note that sugar concentrations depended heavily on pretreatment conditions but not so much on the biocatalyst load used, possibly as a consequence of the small difference between the two biocatalyst loads tested.Thus, for the raw material the final concentrations of total reducing sugars (TRS) were 7.25 ± 1.05 *g*/L (10 FPU/*g* WIS) and 8.52 ± 0.37 *g*/L (15 FPU/*g* WIS) while with the solid accomplished from P11 pretreatment the concentrations were 17.79 ± 0.67 *g*/L (10 FPU/*g* WIS) and 18.62 ± 0.11 *g*/L (15 FPU/*g* WIS). The maximum glucose concentration (10.95 ± 0.07 *g*/L) was obtained using an enzyme load of 15 FPU/*g* WIS and the solid from P10 pretreatment. Enzymatic digestibility, glucose yield (*Y*_Glu/RM_), and total reducing sugars yield (*Y*_TRS/RM_) for raw material and pretreated solids are presented in Table 4. These parameters were calculated using Eqs. 2, 3, and 4, respectively. For the enzymatic digestibility of solids (*ED*), minimum values (8.5% and 9.5%) were observed during the hydrolysis of raw material with enzyme loads of 10 FPU/*g* WIS and 15 FPU/*g* WIS, respectively. In contrast, pretreated solids exhibited *ED* values of 43.5% (P11 with 10 FPU/*g* WIS) and 46.3% (P10 with 15 FPU/*g* WIS). These data imply an increase of about 400% in the enzymatic digestibility of pretreated cellulose compared to the original polymer. Earlier studies on the enzymatic hydrolysis of different biomasses pretreated with diluted sulphuric acid have also reported similar *ED* values: 47.5% for almond prunings pretreated at 220 ℃ for 5 min (Cuevas et al. 2014) and 43.4% for coffee cut-stems pretreated at 120 ℃ for 180 min (Solarte-Toro et al. 2020). The fact that acid pretreatment does not allow for the complete hydrolysis of pretreated cellulose may be due to various factors, such as the high lignin content in WIS, the inhibition of the catalyst by reaction products (cellobiose and glucose), etc. In relation to the presence of lignin, it is interesting to highlight that more severe pretreatments slightly increased the AIL content in the pretreated biomasses (Table 2), simultaneously leading to an improvement in enzymatic action. This could be explained by assuming that acid attack facilitates cellulase access to the substrate by increasing the solids porosity. However, at the same time, non-productive adsorption between the residual lignin and enzyme might occur, reducing the available amount of biocatalyst. In this regard, some authors reported that the solid resulting from the pretreatment of bamboo with diluted sulphuric acid at 160 ℃ for 1 h exhibited an enzymatic digestibility of 29.4%, a value that increased to 64.6% when adding the surfactant PEG 4000 to reduce cellulase-lignin bonds (Huang et al. 2022). In that study, enzyme loadings of 20 FPU/*g* glucan were used, which are higher than those employed in the present work.

The *Y*_Glu/RM_ values (Table 5) were in the range of 3.03–11.68 *g* glucose/100 *g* raw material. The lowest value was observed for the raw almond pruning hydrolysed with an enzyme load of 10 FPU/*g* WIS, whereas the highest value was obtained when the enzymes acted on biomass derived from pretreatment P10 using a load of 15 FPU/*g* WIS (Table 5). In the latter case, the value achieved is equivalent to a glucose production of 29.09 *g* per 100 *g* of WIS. These results indicate that the conditions of acid pretreatment had a strong impact on the enzymatic action. In addition, a slight increase in *Y*_Glu/RM_ was observed as the enzyme load increased from 10 FPU/*g* WIS to 15 FPU/*g* WIS. On the other hand, Table 4 also showed an important difference between the yields of glucose and total reducing sugars, even in experiments where the WIS is virtually devoid of hemicelluloses. For example, after pretreatment P11, enzymatic hydrolysis performed with 15 FPU/*g* WIS led to values of *Y*_Glu/RM_ and *Y*_TRS/RM_ of 11.30 *g* and 19.65 *g*/100 *g* raw material, respectively.

**Table 5:** Glucose and total reducing sugars yields obtained from enzymatic hydrolysis

Run	10 FPU/*g* WIS			15 FPU/*g* WIS		
	*ED* (%)	*Y*_Glu/RM_	*Y*_TRS/RM_	*ED* (%)	*Y*_Glu/RM_	*Y*_TRS/RM_
RM	8.54 ± 1.86	3.03 ± 0.66	7.25 ± 1.05	9.50 ± 0.80	3.37 ± 0.28	8.52 ± 0.37
P1	19.13 ± 0.02	6.01 ± 0.01	11.64 ± 1.32	22.94 ± 0.59	7.21 ± 0.19	12.54 ± 1.85
P2	20.81 ± 2.31	6.51 ± 0.72	11.56 ± 0.90	23.40 ± 0.43	7.32 ± 0.13	11.62 ± 0.04
P3	29.02 ± 1.75	8.31 ± 0.50	13.39 ± 1.73	27.63 ± 1.05	7.91 ± 0.30	12.38 ± 0.11
P4	27.85 ± 2.19	8.07 ± 0.63	13.67 ± 2.36	28.06 ± 1.74	8.13 ± 0.50	13.63 ± 0.31
P5	30.57 ± 0.19	8.53 ± 0.05	13.93 ± 0.07	32.93 ± 0.36	9.19 ± 0.10	17.09 ± 1.64
P6	30.71 ± 0.86	8.57 ± 0.24	13.98 ± 0.56	31.25 ± 6.37	8.87 ± 1.81	15.22 ± 0.75
P7	33.27 ± 5.04	9.35 ± 1.51	14.86 ± 1.12	33.87 ± 1.65	9.51 ± 0.56	16.64 ± 1.40
P8	36.00 ± 1.26	10.18 ± 0.36	16.29 ± 1.25	34.95 ± 1.72	9.88 ± 0.49	16.05 ± 0.43
P9	38.71 ± 0.44	10.57 ± 0.12	16.14 ± 1.00	37.78 ± 0.72	10.32 ± 0.20	15.62 ± 0.23
P10	42.98 ± 0.57	10.84 ± 0.14	16.40 ± 0.08	46.31 ± 0.32	11.68 ± 0.08	16.53 ± 0.61
P11	43.05 ± 0.07	11.54 ± 0.02	18.78 ± 0.50	42.16 ± 0.61	11.30 ± 0.16	19.65 ± 0.11

RM: Assay carried out with raw material. P1-P11: Assays carried out with pretreated solids (WIS). *ED*: Enzymatic digestibility, or g glucose by enzymatic hydrolysis/100 *g* glucose in substrate. *Y*_Glu/RM_: g glucose by enzymatic hydrolysis/100 *g* raw material. *Y*_TRS/RM_: g total reducing sugars by enzymatic hydrolysis/100 *g* raw material

**Fig. 4:**
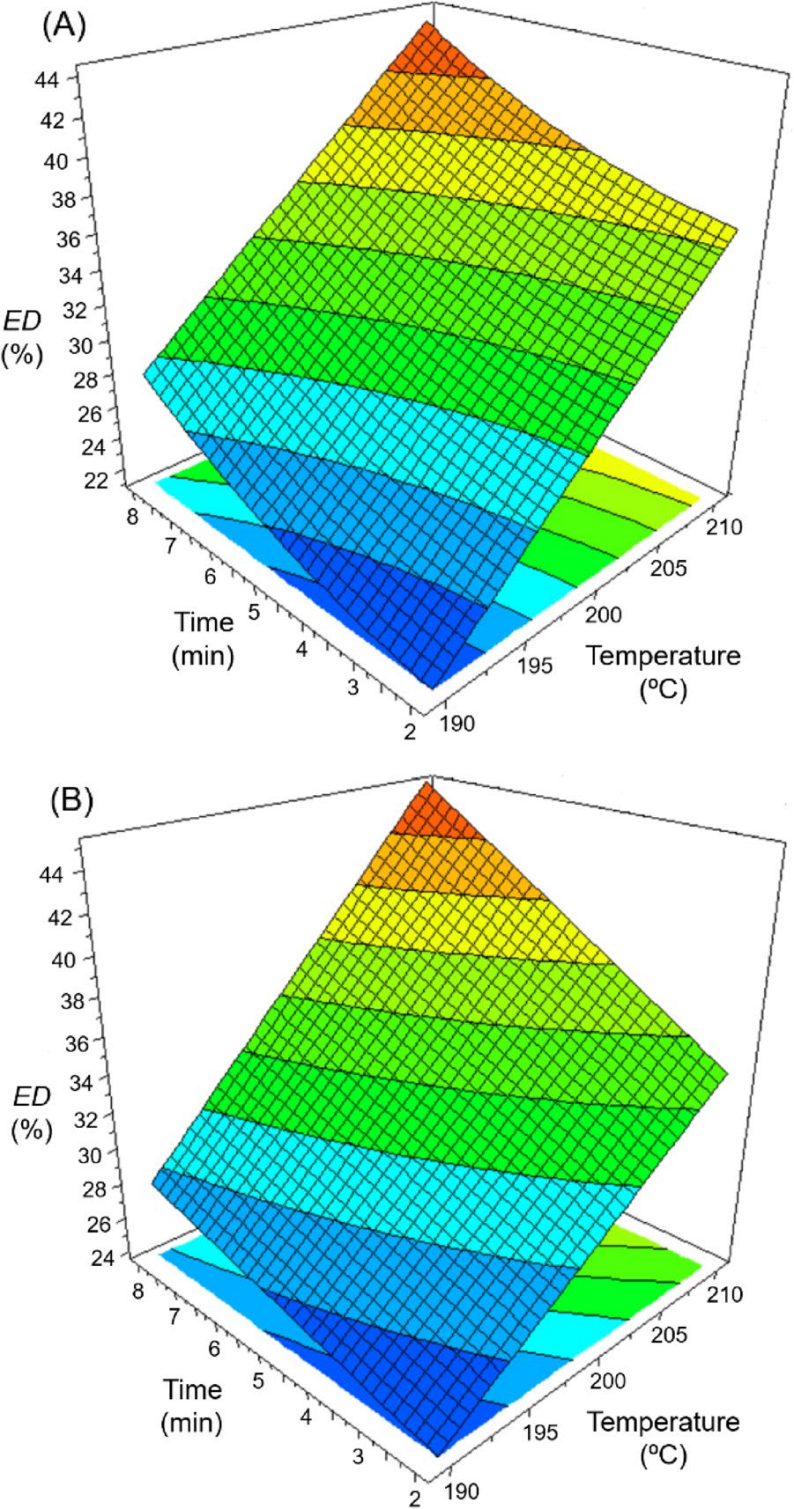
Response surface plots representing the effect of temperature and pretreatment time on enzymatic digestibility (*ED*) of the pretreated cellulose at two enzyme loadings: 10 FPU/*g* WIS (**A**) and 15 FPU/*g* WIS (**B**)

The relationship between pretreatment conditions (temperature and reaction time) and the enzymatic digestibility of WIS could be described mathematically by Eq. (1). A value of *R*^2^ = 0.988 was obtained for 10 FPU/*g* WIS with the following a_i_ values: 31.182 ± 0.436 (a_1_), 8.018 ± 0.367 (a_2_), 3.466 ± 0.367 (a_3_) and 1.138 ± 0.418 (a_5_). Whereas for 15 FPU/*g* WIS, a *R*^2^ = 0.990 was achieved with 32.844 ± 0.266 (a_1_), 7.123 ± 0.312 (a_2_), 3.797 ± 0.312 (a_3_) and 1.675 ± 0.442 (a_6_). From the above values, response surface plots could be represented (Fig. 4). These figures showed that the enzymatic digestibility of WIS is strongly affected by the pretreatment with diluted acid, in such a way that the maximum enzymatic digestibility is obtained using the most severe pretreatments. For example, for enzymatic hydrolyses performed with a load of 15 FPU/*g* WIS, the maximum *ED* value (45.4%) was reached after a pretreatment carried out at 210 ℃ for 8 min (Fig. 4B).

### Optimisation of the sugar production process and mass balance

In order to study the impact of temperature and pretreatment time on the overall production of fermentable sugars, the sum of yields of glucose, xylose, arabinose, galactose, and mannose obtained in acid pretreatments (*Y*_TMS_ in Table 3) was combined with the glucose yield from enzymatic hydrolysis (*Y*_Glu/RM_ in Table 5).This resulted in a global monosaccharide yield from the overall process, *Y*_global_), a parameter that ranged between 18.68% and 31.37%. By applying the *Y*_*global*_ parameter as response in Modde 6.0, data in Table 6 were generated. This table contains the most relevant information about the two mathematical models describing the dependence of the overall production of monosaccharides with pretreatment conditions for enzymatic loads of 10 FPU/*g* WIS and 15 FPU/*g* WIS. Table 6 revealed high *R*^*2*^ values in both models, as well as the existence of quadratic terms for factors *T*_R_ and *t*_R_.

**Table 6:** Model parameters (a_i_), standard errors (SE), and significance level (*p*) for the mathematical model to describe (*Y*_*global*_)*

Response variable	a_i_	SE	*p*-value (Prob > *F*)	*R*^2^	*R*^2^_adjust_
*Y*_global_ (A)	a_0_: 30.823	0.407	3.54·10^–10^	0.984	0.974
	a_1_: –2.224	0.249	1.10·10^–4^		
	a_2_: –1.508	0.249	9.17·10^–4^		
	a_3_: –4.784	0.296	3.59·10^–6^		
	a_4_: –1.936	0.296	6.15·10^–4^		
*Y*_global_ (B)	a_0_: 31.197	0.307	6.08·10^–11^	0.992	0.986
	a_1_: –2.647	0.188	7.96·10^–6^		
	a_2_: –1.434	0.188	2.63·10^–4^		
	a_3_: –4.802	0.224	6.65·10^–7^		
	a_4_: –2.168	0.224	6.89·10^–5^		

^*^Y_global_: Overall monosaccharides yield of the process for enzymatic hydrolysis carried out with biocatalyst loads of 10 FPU/*g* WIS (**A**) and 15 FPU/*g* WIS (**B**)

**Fig. 5:**
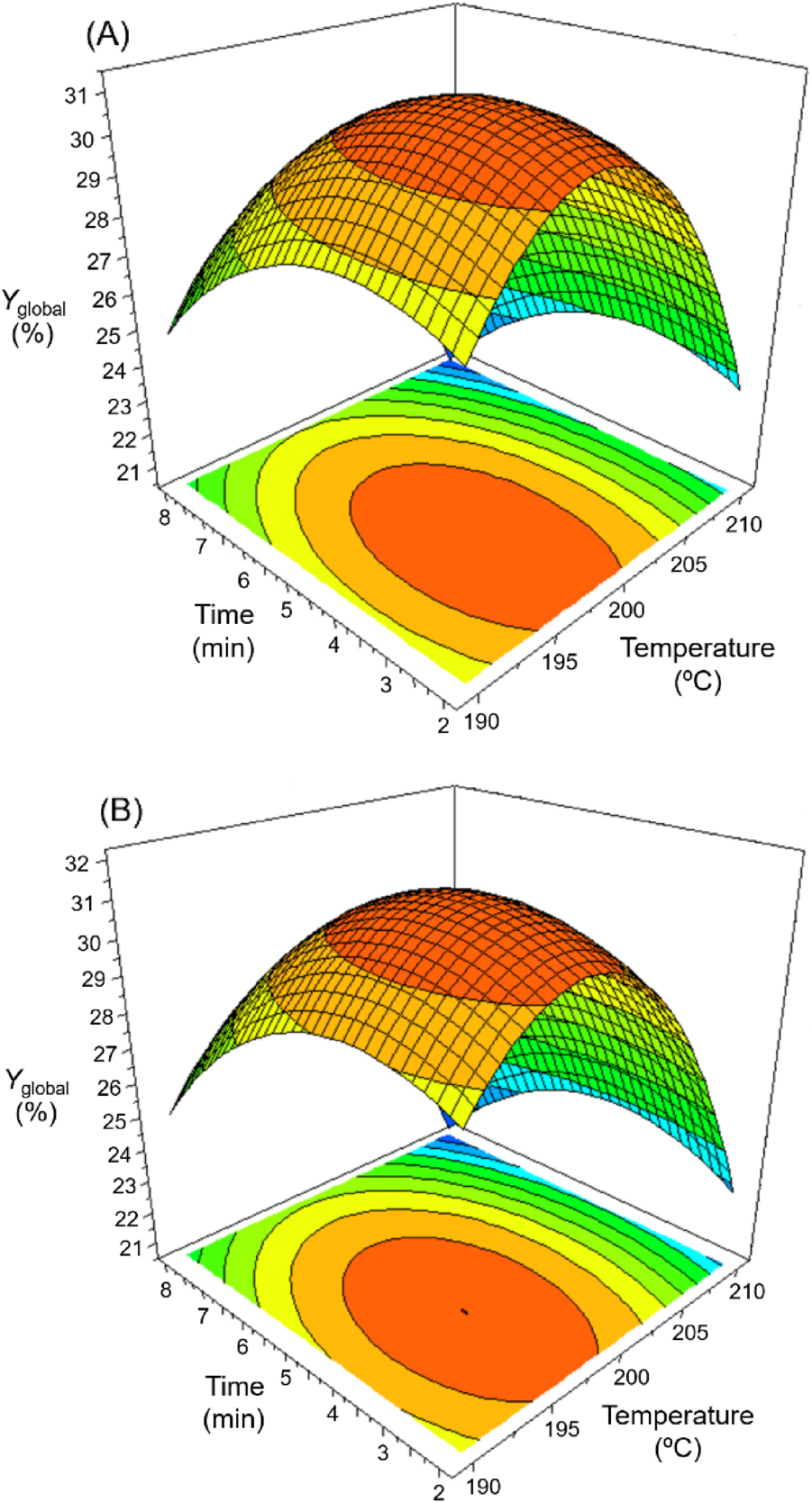
Response surface plots representing the effect of temperature and pretreatment time on the global yield of monomeric sugars (*Y*_global_) from almond prunings using two enzymatic loadings of cellulase: 10 FPU/*g* WIS (**A**) and 15 FPU/*g* WIS (**B**)

Response surface plots representing the effect of *T*_R_ and *t*_R_ on *Y*_global_ are shown in Fig. 5. This helped in identifying the maximum values of *Y*_global_ obtained under the studied pretreatment conditions. Thus, for enzymatic hydrolyses with Celluclast 1.5 L (loads equal to 10 FPU/*g* WIS) the maximum *Y*_global_ value (31.37%) was accomplished with pretreatment conditions of 197.6 ℃ and 3.8 min, while for enzyme loads of 15 FPU/*g* WIS, the maximum value of *Y*_global_ (31.80%) was achieved with a pretreatment at 197.2 ℃ and 4.0 min. The pretreatment conditions reached for the two enzyme load series were very close. So, from a practical point of view, the temperature of 197 ℃ and the time of 4.0 min can be adopted as appropriate values to maximise the production of sugars from almond tree pruning. Cara et al. (2008) achieved a maximum *Y*_global_ value of 36.3% by pretreating olive prunings with diluted sulphuric acid (1%) and then subjecting the WIS to enzymatic hydrolysis with a mixture of Celluclast 1.5 L (15 FPU/*g* substrate) and Novozym 188 (15 International Unit/*g* substrate). The above value is slightly higher than that achieved in the present study but implies the use of a higher enzymatic load.

**Fig. 6:**
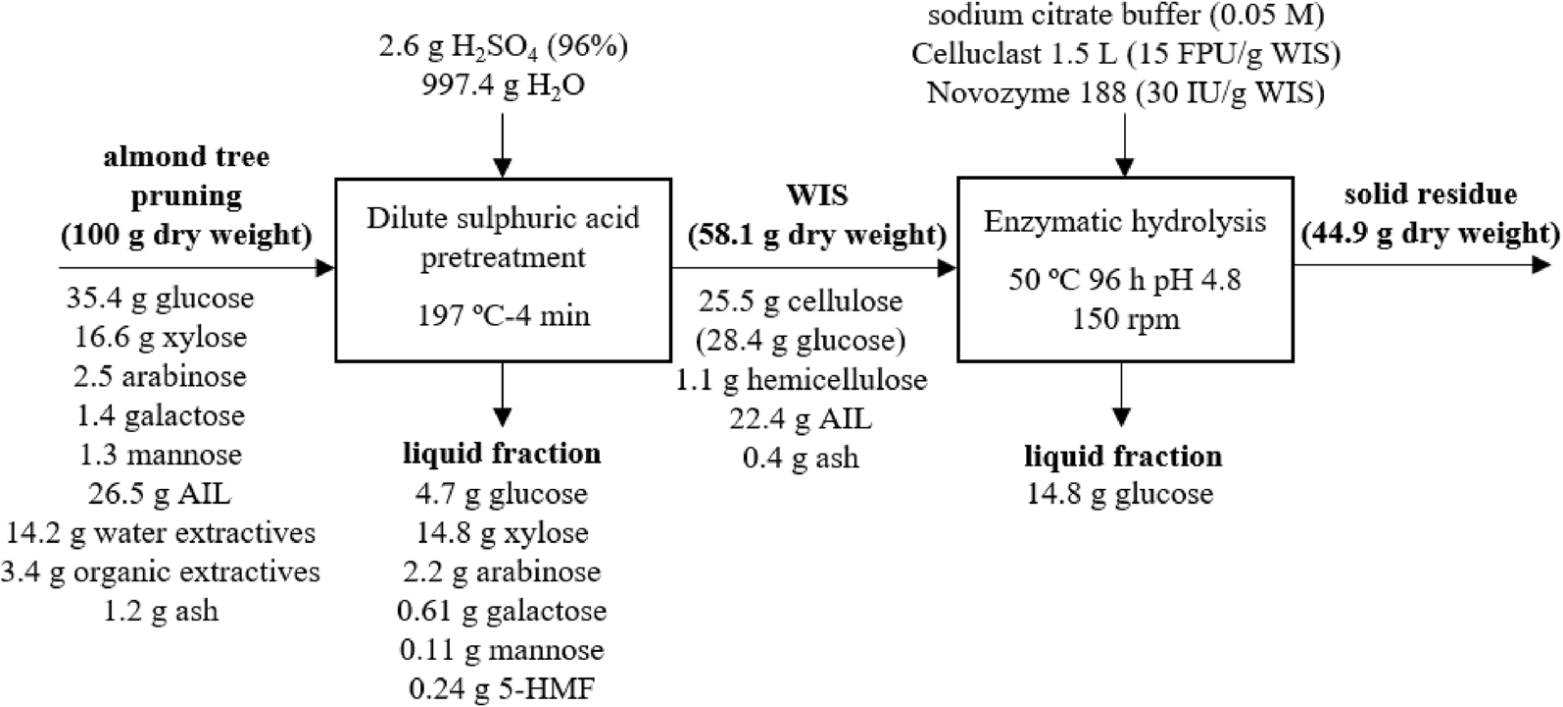
Material balance flow of the H_2_SO_4_ pretreatment of almond-tree pruning biomass, under optimal conditions, and the subsequent enzyme hydrolysis of pretreated solids

To study the effect of the incorporation of the enzyme complex “Novozym 188” in the enzymatic production of glucose, the raw material was pretreated under previously optimised conditions (197 ℃-4 min), and the WIS was hydrolysed with Celluclast 1.5 L (15 FPU/*g* WIS) supplemented with Novozym 188 (30 IU/*g* WIS). By this method, an *ED* value of 52.1% was reached, which was equivalent to the production of 14.77 *g* of glucose by enzymatic hydrolysis per 100 *g* of raw material. The addition of Novozym188 increased the β-glucosidase activity which led to a *Y*_global_ value of 36.8%. This data is equivalent to a recovery of 64.3% of the sugars present in the raw material. The above *Y*_global_ values are in line with earlier research findings related to high-temperature pretreatments followed by enzymatic hydrolysis of various biomasses: 37.8% using rapeseed straw (Romero et al. 2018) and 37% using olive tree biomass (López-Linares et al. 2013). Figure 6 shows the material balance of the sugar production process from almond tree pruning biomass including the pretreatment with diluted sulphuric acid under optimal conditions (197 ℃-4 min) and the subsequent enzymatic hydrolysis of WIS using Celluclast 1.5 L (15 FPU/*g* WIS) and Novozym 188 (30 IU/*g* WIS). The results obtained under optimal conditions also confirmed the validity of the mathematical models used in the present work (interval confidence of 95%).

### Characteristics of biomass and solid fractions for thermochemical applications

**Fig. 7:**
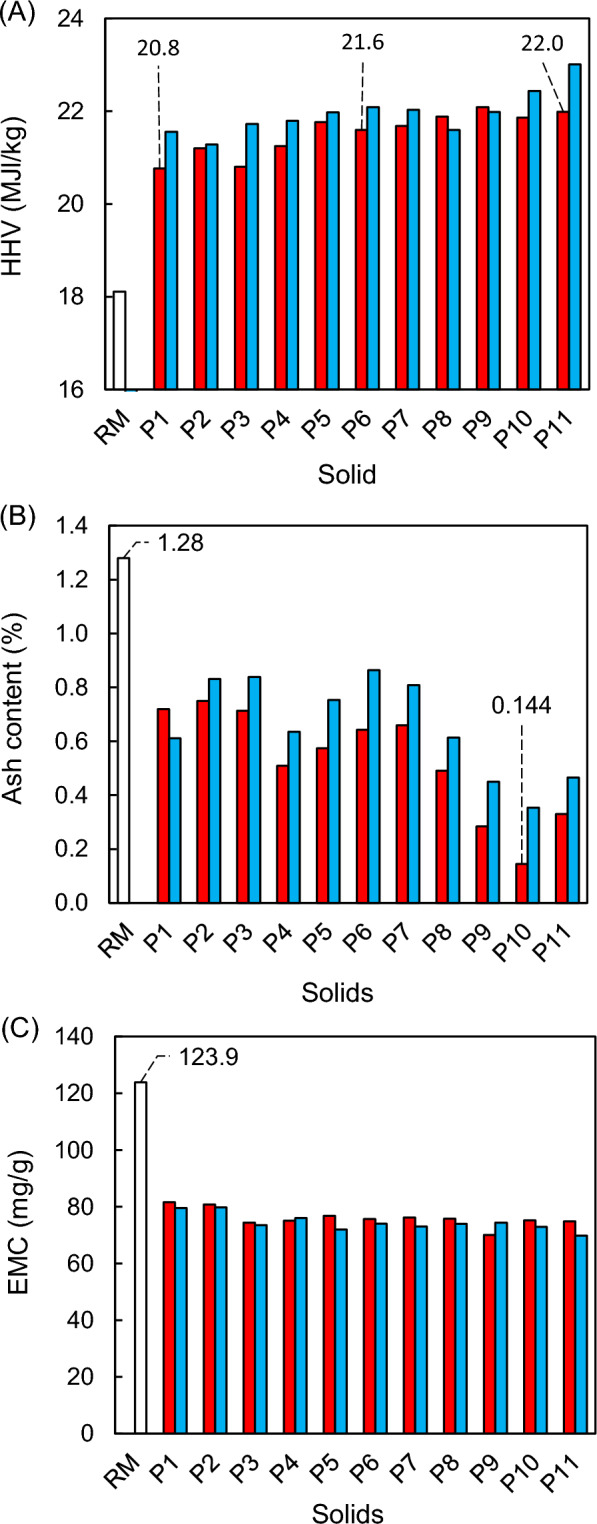
Higher Heating Value (**A**), ash percentage (**B**) and Equilibrium Moisture Content (**C**) for the raw material (white bars), WIS (red bars) and resulting solids from enzymatic hydrolysis of WIS (blue bars)

The diluted acid pretreatment of almond pruning biomass, followed by enzymatic hydrolysis of the pretreated solids, produces both liquid and solid fractions. The liquid fractions are generally used for the recovery of monosaccharides. But it is also essential to valorise the final solid from the sugar production process (a rich-lignin solid residue) to achieve an integral use of the raw material. Thermochemical utilisation of biomass is generally favoured by increasing higher heating value (HHV) and reducing both the percentage of ash and the Equilibrium Moisture Content (EMC). The EMC is indicative of the capacity of adsorption of moisture by a solid under certain environmental conditions and, in this work, it has been expressed as mg of water adsorbed in each gram of dry solid (mg/*g*). Figure 7 shows the values of the aforementioned three parameters for the raw material, the solids resulting from acid pretreatments (WIS) and the solids resulting from the enzymatic hydrolysis of WIS using an enzyme load of 15 FPU/*g* WIS. With respect to the higher heating values (Fig. 7A), the raw material had an HHV of 18.11 ± 0.1 MJ/kg, a value clearly lower than that of the solids generated in acid pretreatments (20.48–22.09 MJ/kg) and enzymatic hydrolyses (21.28–23.01 MJ/kg,). In general, the application of pretreatments with higher temperatures and reaction times led to an increase in the HHV of the WIS. This way, solids derived from pretreatments P1, P6, and P11 had HHV values of 20.77 MJ/kg, 21.60 MJ/kg and 21.99 MJ/kg, respectively (Fig. 7A). This could be due to the increase in the percentage of AIL in pretreated solids (Table 2), as lignin is the structural component of biomass with the highest heating value (Maksimuk et al. 2021). This fact would also explain how, in general, the solids resulting from the enzymatic hydrolysis showed slightly higher heating values than WIS, as the enzymes reduced the amount of cellulose available in the solids without altering the lignin fraction. The maximum HHV reached in the experimental study (5505 kcal/kg) was achieved with the solid that remained after the pretreatment P11 (214.1 ℃-5 min) following by the enzymatic hydrolysis of the corresponding WIS. This HHV value represents an increase of 27% over the heating value of the raw material, and it implies a higher energy densification of almond wood than that reported by Aguado et al. (2020) when the same type of biomass was subjected to wet torrefaction at 250 ℃-10 min (23% increase) and at 225 ℃-60 min (26% increase), i.e. applying much more energetically intensive treatments. On the other hand, Fig. 7B shows that the ash content of the WIS (0.14%–0.75%) was clearly lower than the original biomass (1.28 ± 0.04%). This could be beneficial for the thermochemical use of the pretreated solids. The effect of acid treatments on the ash content of lignocellulosic biomasses has been studied by different authors (Lee et al. 2021) and could be explained by considering that the H^+^ ion reacts with the alkali components in the biomass via neutralisation reactions (Chin et al. 2015). Enzymatic hydrolysis generated solids with ash percentages slightly higher than those of hydrolysed substrates (0.35%–0.86%). The reason could be that lignin contains more inorganic material than cellulose. Regarding the moisture adsorption capacity (Fig. 7C) of the different biomasses under a constant relative humidity atmosphere of 75.5%, the raw material showed an EMC value of 123.9 mg/*g*, much higher than that of the WIS (81.6–70.1 mg/*g*) and the solids from enzymatic hydrolysis (79.8–69.9 mg/*g*). These results could be due to the more hydrophobic nature of lignin compared to cellulose or hemicellulose (Piao et al. 2010). In the case of the process scheme developed under optimised conditions for the production of sugars (Fig. 6), it was found that the residual solid generated after enzymatic hydrolysis had values of HHV, ash percentage, and EMC of 5259 ± 102 kcal/kg (21.4% higher than raw material), 0.87 ± 0.04% (32.0% lower than raw material), and 72.1 ± 4.3 (41.8% lower than raw material), respectively.

## Conclusions

Almond-tree pruning biomass is an agricultural waste from which sugars can be obtained by applying the process scheme tested in this work combined with a response surface methodology. In this way, pretreatment conditions have been established to maximise the extraction of hemicellulose sugars in the prehydrolysate (195.7 ℃-3.5 min), the enzymatic digestibility of pretreated cellulose (210.0 ℃-8.0 min), and the overall sugar production (197.0 ℃-4.0 min). Globally, 36.8 kg of monosaccharides were reached from 100 kg of almond prunings, which represents an improvement in the yield and operating conditions compared to previously published data. On the other hand, the hydrolytic treatments of almond prunings generate final solid residues with improved characteristics for thermochemical use (higher HHV and lower ash contents and moisture adsorption capacities).

## Data Availability

Not applicable.
